# AMP-activated protein kinase complexes containing the β2 regulatory subunit are up-regulated during and contribute to adipogenesis

**DOI:** 10.1042/BCJ20180714

**Published:** 2019-06-26

**Authors:** Omar J. Katwan, Fatmah Alghamdi, Tarek A. Almabrouk, Sarah J. Mancini, Simon Kennedy, Jonathan S. Oakhill, John W. Scott, Ian P. Salt

**Affiliations:** 1 Institute of Cardiovascular and Medical Sciences, College of Medical, Veterinary and Life Sciences, University of Glasgow, Glasgow G12 8QQ, U.K.; 2 Department of Biochemistry, College of Medicine, University of Diyala, Baqubah, Iraq; 3 Faculty of Medicine, King Abdulaziz University, Jeddah, Kingdom of Saudi Arabia; 4 Medical School, University of Zawia, Zawia, Libya; 5 St Vincent's Institute of Medical Research and Department of Medicine, University of Melbourne, Fitzroy, VIC 3065, Australia; 6 Mary MacKillop Institute for Health Research, Australian Catholic University, 215 Spring Street, Melbourne 3000, Australia; 7 The Florey Institute of Neuroscience and Mental Health, Parkville 3052, Australia

**Keywords:** adipocytes, adipogenesis, AMPK

## Abstract

AMP-activated protein kinase (AMPK) is a heterotrimer of α-catalytic and β- and γ-regulatory subunits that acts to regulate cellular and whole-body nutrient metabolism. The key role of AMPK in sensing energy status has led to significant interest in AMPK as a therapeutic target for dysfunctional metabolism in type 2 diabetes, insulin resistance and obesity. Despite the actions of AMPK in the liver and skeletal muscle being extensively studied, the role of AMPK in adipose tissue and adipocytes remains less well characterised. Small molecules that selectively influence AMPK heterotrimers containing specific AMPKβ subunit isoforms have been developed, including MT47-100, which selectively inhibits complexes containing AMPKβ2. AMPKβ1 and AMPKβ2 are the principal AMPKβ subunit isoforms in rodent liver and skeletal muscle, respectively, yet the contribution of specific AMPKβ isoforms to adipose tissue function, however, remains largely unknown. This study therefore sought to determine the contribution of AMPKβ subunit isoforms to adipocyte biology, focussing on adipogenesis. AMPKβ2 was the principal AMPKβ isoform in 3T3-L1 adipocytes, isolated rodent adipocytes and human subcutaneous adipose tissue, as assessed by the contribution to total cellular AMPK activity. Down-regulation of AMPKβ2 with siRNA inhibited lipid accumulation, cellular adiponectin levels and adiponectin secretion during 3T3-L1 adipogenesis, whereas down-regulation of AMPKβ1 had no effect. Incubation of 3T3-L1 cells with MT47-100 selectively inhibited AMPK complexes containing AMPKβ2 whilst simultaneously inhibiting cellular lipid accumulation as well as cellular levels and secretion of adiponectin. Taken together, these data indicate that increased expression of AMPKβ2 is an important feature of efficient adipogenesis.

## Introduction

AMP-activated protein kinase (AMPK) is the downstream component of a protein kinase cascade that responds to increases in AMP (or ADP) relative to ATP, resulting from such stimuli as hypoxia, nutrient deprivation and ischaemia. AMPK acts to stimulate ATP synthesis and attenuate ATP consumption, thereby maintaining energy balance [1–4]. Mammalian AMPK is a heterotrimer of an α-catalytic subunit and regulatory β- and γ-subunits, in which the binding of AMP to the γ-subunit allosterically activates AMPK whilst also promoting activating phosphorylation of the α-subunit at Thr172 by liver kinase B1 (LKB1), effects antagonised by ATP [1,2]. The β-subunit contains a C-terminal domain that forms the core of the heterotrimeric complex, in addition to a carbohydrate-binding domain of poorly defined function [1,2]. There are two isoforms of the α- and β-subunits and three isoforms of the γ-subunit, which exhibit differential tissue expression [5,6]. Furthermore, a number of direct AMPK activators, such as A769662 and compound 991, have been developed, which have been demonstrated to bind a site between the β-subunit carbohydrate-binding domain and the α-subunit [7]. Compounds binding at this site show marked selectivity for AMPK complexes containing β1 rather than β2 [8], such that pharmacological targeting of specific tissues based on their AMPKβ isoform expression is feasible.

In addition to the important role that AMPK plays in maintaining cellular energy metabolism, it is clear that AMPK also contributes to the coordination of nutrient metabolism in the whole organism, with activated AMPK inhibiting multiple pathways including lipogenesis, cholesterol synthesis, protein translation, hepatic gluconeogenesis whilst stimulating fatty acid oxidation, feeding and striated muscle glucose uptake. As a consequence, AMPK activation has attracted interest as a potential therapeutic target for dysfunctional metabolism in insulin resistance, type 2 diabetes and obesity [1–4]. Indeed, recent studies have reported that a systemic pan-AMPK activator improves glucose homeostasis in preclinical models [9] and that an AMPK activator targeting complexes containing AMPKβ1corrects non-alcoholic fatty liver disease (NAFLD) in primate preclinical models [10]. Despite the well-characterised actions of AMPK in muscle and liver, the role of AMPK in adipose tissue and adipocytes remains less well characterised. AMPK stimulation in response to activators or physiological stimuli such as fasting, exercise and catecholamines is associated with reduced lipogenesis and insulin-stimulated glucose uptake, increased fatty acid oxidation and mitochondrial biogenesis, anti-inflammatory signalling and altered adipocytokine secretion in cultured or primary isolated adipocytes and white adipose tissue (WAT) [3,4]. Although the precise role of AMPK remains uncertain, AMPK activation is associated with altered adipose tissue lipolysis [3,4] and AMPK activation has an important role in generating and maintaining brown adipose tissue (BAT). In support of an important role for AMPK in adipose tissue metabolism, AMPK activity is reduced in WAT from obese and insulin-resistant rodents and humans [11–13].

A number of studies have demonstrated that incubation with AMPK activators inhibited differentiation of 3T3-L1 preadipocytes or human mesenchymal stem cells into adipocytes *in vitro* [14–20] an effect phenocopied by overexpression of AMPKα or LKB1 [21,22]. In keeping with an anti-adipogenic action of AMPK, down-regulation of AMPK with siRNA has been reported to increase lipid accumulation and markers of adipogenesis in 3T3-L1 preadipocytes and human mesenchymal stem cells [23–27] and silencing of the activating AMPK Thr172 kinases LKB1 or Ca^2+^-calmodulin-dependent kinase kinase-2 (CaMKK2) also increases adipogenesis of 3T3-L1 preadipocytes [22,28,29]. These data suggest AMPK activation contributes to suppression of lipogenesis and adipogenesis, yet it remains unclear as to the precise role of AMPK subunit isoforms in adipogenesis. In rodents, AMPKβ1 and AMPKβ2 are the predominant AMPKβ isoforms in liver and muscle, respectively, yet the role of AMPKβ isoforms in adipose tissue remains poorly characterised [3–5]. This study therefore sought to determine the contribution of AMPKβ subunit isoforms to adipogenesis in 3T3-L1 adipocytes.

## Materials and methods

### Materials

DMEM, FCS, newborn calf serum (NCS), penicillin/streptomycin, trypsin and mouse anti-GAPDH (#AM4300) antibodies were purchased from Invitrogen (Paisley, U.K.). Rabbit anti-ACC (acetyl-CoA carboxylase, #3676), anti-phospho-ACC S79 (#3661), anti-AMPKα (#2532), anti-phospho-AMPKα T172 (#2535), anti-AMPKβ1 (#4182), anti-AMPKβ2 (#4148), anti-AMPKβ1/β2 (#4150), anti-C/EBPα (CCAAT-enhancer-binding protein-α, #8178), anti-LKB1 (#3047), anti-perilipin-1 (#9349) and anti-PPARγ (peroxisome proliferator-activated receptor-γ, #2443) antibodies were purchased from Cell Signaling Technology (New England Biolabs, Hitchin, U.K.). Sheep anti-AMPKα1, anti-AMPKα2, anti-AMPKβ1 and anti-AMPKβ2 antibodies used for immunoprecipitation of AMPK complexes containing specific subunit isoforms (or immunoblotting in the case of anti-AMPKα1 and anti-AMPKα2 antibodies) were a kind gift from Professor D.G. Hardie (University of Dundee) and have been described previously [30–32]. Donkey Infrared dye-labelled secondary antibodies and REVERT total protein stain were from LI-Cor Biosciences (Cambridge, U.K.). Rabbit anti-adiponectin antibody was a kind gift from Professor G.W. Gould (University of Glasgow) and has been described previously [33]. Ponceau-S, oil red O and Mayers haematoxylin were purchased from Sigma–Aldrich Ltd (Gillingham, U.K.). Type 1 collagenase was from Worthington Biochemical Corp. (Lakewood, NJ, U.S.A.). MT47-100 was synthesised as described previously [34]. Lipofectamine 2000 and siRNA targeted to AMPKβ1 (#s72125, #s72126) or AMPKβ2 (#s99128, #s99130) as well as scrambled siRNA were purchased from Fisher Scientific UK Ltd (Loughborough, U.K.). Mouse adiponectin Quantikine ELISA kits were purchased from R&D systems (Minneapolis, MN, U.S.A.). All other reagents were from sources described previously[20,35].

### Human adipose tissue

The human adipose tissue lysates were those previously described [35], prepared from gluteal adipose tissue biopsies of men aged 50–70 years with type 2 diabetes (duration > 6 months) and had been stored at −80°C prior to AMPK isoform assay analyses. The North Glasgow University Hospitals National Health Service Trust Ethics Committee approved the study, and all individuals gave written informed consent.

### Isolation of rodent adipocytes

All animals were housed in a 12-h light dark cycle with access to food and water *ad libitum*. All experimental procedures were carried out in accordance with the United Kingdom Animal Procedures Act (1986) and with the ‘Guide for the Care and Use of Laboratory Animals' published by the US National Institutes of Health (eighth edition) at the University of Glasgow. Mesenteric and epididymal adipose tissues were rapidly excised from male 8–12-week-old sv129 mice or 200–250** **g male Sprague–Dawley rats immediately after being killed and placed in pre-warmed (37°C) collection buffer (128** **mmol/l NaCl, 4.7** **mmol/l KCl, 5** **mmol/l NaH_2_PO_4_, 1.2** **mmol/l MgSO_4_, 20** **mmol/l HEPES-NaOH, 1.5** **mmol/l CaCl_2_, 1% (w/v) BSA, 10 µmol/l adenosine, 3** **mmol/l glucose). Adipose tissue depots were weighed and 4 vol. of collection buffer containing type 1 collagenase (2** **g/l) added. The tissue was chopped with scissors and incubated in a shaking water bath at 37°C for 15** **min. Adipocytes were sieved into a warmed collection vial and washed by floatation with collection buffer (three times) and collection buffer without BSA (two times). Livers were excised from male 8–12-week-old sv129 mice and snap-frozen in liquid N_2_. Livers were homogenised by 20 passes in a Dounce homogeniser in 4 vol. IP buffer [50** **mmol/l Tris–HCl, pH 7.4 at 4°C, 150** **mmol/l NaCl, 50** **mmol/l NaF, 1** **mmol/l Na_4_P_2_O_7_, 1** **mmol/l EDTA, 1** **mmol/l EGTA, 1% (v/v) Triton-X-100, 1% (v/v) glycerol, 1** **mmol/l dithiothreitol, 1** **mmol/l Na_3_VO_4_, 0.1** **mmol/l benzamidine, 0.1** **mmol/l phenylmethylsulphonyl fluoride, 5** **mg/l soybean trypsin inhibitor] and sequential polyethylene glycol (PEG) precipitation was used to prepare 2.5–6.25% PEG precipitates. PEG precipitates were resuspended in IP buffer prior to AMPK assay.

### 3T3-L1 cell culture

3T3-L1 preadipocytes (from ATCC, mycoplasm-free) were cultured in DMEM supplemented with 10% (v/v) NCS. Differentiation into adipocytes was initiated 2 days post-confluence when the culture medium was replaced with DMEM supplemented with 10% (v/v) FCS, 0.5** **mmol/l 3-isobutyl-1-methylxanthine (IBMX), 0.25 µmol/l dexamethasone, 1** **mg/l porcine insulin and 5 µmol/l troglitazone. After 3 days, the medium was replaced with DMEM supplemented with 10% (v/v) FCS, 5 µmol/l troglitazone and 1** **mg/l insulin. After a further 3 days the medium was replaced with DMEM supplemented with 10% (v/v) FCS. Adipocytes were used for experimentation 8–12 days post-induction of differentiation. In experiments investigating the effect of AMPKβ isoform down-regulation or MT47-100 on adipogenesis, 3T3-L1 preadipocytes were differentiated to adipocytes in the absence of troglitazone.

### Preparation of 3T3-L1 cell lysates

3T3-L1 preadipocytes/adipocytes were incubated in serum-free DMEM for 2 h prior to lysis in lysis buffer [50 mmol/l Tris–HCl, pH 7.4 at 4°C, 50 mmol/l NaF, 1 mmol/l Na_4_P_2_O_7_, 1 mmol/l EDTA, 1 mmol/l EGTA, 1% (v/v) Triton-X-100, 250 mmol/l mannitol, 1 mmol/l dithiothreitol, 1 mmol/l Na_3_VO_4_, 0.1 mmol/l benzamidine, 0.1 mmol/l phenylmethylsulphonyl fluoride, 5 mg/l soybean trypsin inhibitor] on ice. For analysis of PPARγ and C/EBPα levels, 3T3-L1 cells cultured in six-well plates were lysed directly in 100 µl sample buffer (50 mmol/l Tris–HCl, pH 6.8, 2% (w/v) SDS, 10% (v/v) glycerol, 0.1% (w/v) bromophenol blue, 50 mmol/l dithiothreitol).

### Immunoprecipitation and assay of AMPK activity

AMPK-specific isoforms were immunoprecipitated from 3T3-L1 preadipocytes and adipocytes, isolated rodent adipocytes, and human subcutaneous adipose tissue using sheep anti-AMPKα1, anti-AMPKα2 [30], anti-AMPKβ1 [31] or anti-AMPKβ2 [32] antibodies. The immunoprecipitates were then assayed for AMPK activity using the SAMS substrate peptide as described previously [20,35].

### SDS–PAGE and immunoblotting

Equal amounts of protein, as determined by Bradford assay, were resolved by SDS–PAGE, transferred to a nitrocellulose membrane and subjected to immunoblotting with the antibodies indicated as described previously [20,35]. Transfer of proteins was assessed by staining of immunoblots with Ponceau stain (0.5% (w/v) Ponceau-S, 1% (v/v) acetic acid). Immunolabelled proteins were visualised using infrared dye-labelled secondary antibodies and an Odyssey Sa infrared imaging system (Li-Cor Biosciences UK Ltd, Cambridge, U.K.). Band density was analysed with Image J software.

### siRNA-mediated knockdown of AMPKβ isoforms

3T3-L1 preadipocytes were seeded in 12-well plates at 6 ×** **10^4^ cells/well in 800** **µl of the culture medium and were allowed to attach overnight. At 70% confluence, the culture medium was replaced with 800** **µl/well fresh culture medium, and 200** **µl/well Opti-MEM I reduced serum medium containing 2** **µl Lipofectamine 2000 and 0.5 µmol/l siRNA (scrambled or targeted to AMPKβ1 or AMPKβ2). After 24** **h, the medium was replaced with normal culture medium, and cells incubated for a further 24** **h prior to differentiation.

### RNA extraction from 3T3-L1 adipocytes and gene expression analysis

RNA was extracted from 3T3-L1 cells 0, 3, and 8 days after the initiation of differentiation using an RNeasy kit (Qiagen). Between 400 and 1000** **ng of RNA was reverse-transcribed using a High Capacity cDNA Reverse Transcription kit (Applied Biosystems). qPCR was performed with an Applied Biosystems ABI-PRISM 7900HT Sequence Detection System. Gene expression was normalised to TATA-binding protein (Tbp) using Assays on Demand and QPCR master mix (Applied Biosystems) as described previously [20]. The following TaqMan^®^ Gene Expression Assays (Applied Biosystems) were used: Tbp (Mm01277042_m1), Prkab1 (AMPKβ1, Mm01201921_m1) and Prkab2 (AMPKβ2, Mm01257133_m1). Relative levels of mRNA were assessed using the ΔΔCt method.

### Oil red O staining of 3T3-L1 adipocytes

3T3-L1 preadipocytes cultured on glass coverslips were incubated in the presence or absence of MT47-100 or siRNA and at various intervals from the initiation of differentiation, cells were fixed with 10% (v/v) formalin for 1** **h. Coverslips were subsequently washed once in 60% (v/v) isopropanol and left to dry completely prior to incubation in 5.142** **mmol/l oil red O, 60% (v/v) isopropanol for 10** **min. Coverslips were then washed four times with distilled water, left to dry and submerged in Mayers haematoxylin (Sigma–Aldrich) for 4** **min followed by 3% (v/v) NH_4_OH for 10 s. Coverslips were mounted onto slides and images captured using AxioVision microscope software (Zeiss, Germany), with the investigator blinded as to the experimental conditions.

### Adiponectin secretion ELISA

Conditioned medium during 3T3-L1 adipogenesis was collected and adiponectin content assayed using a mouse adiponectin Quantikine ELISA kit according to the manufacturer's recommended protocol.

### Statistical analysis

Results are expressed as mean ± SEM. Statistically significant differences were determined using a two-tailed *t*-test or one- or two-way ANOVA where appropriate, with *P* < 0.05 as significant using GraphPad Prism software.

## Results

**Figure 1.**
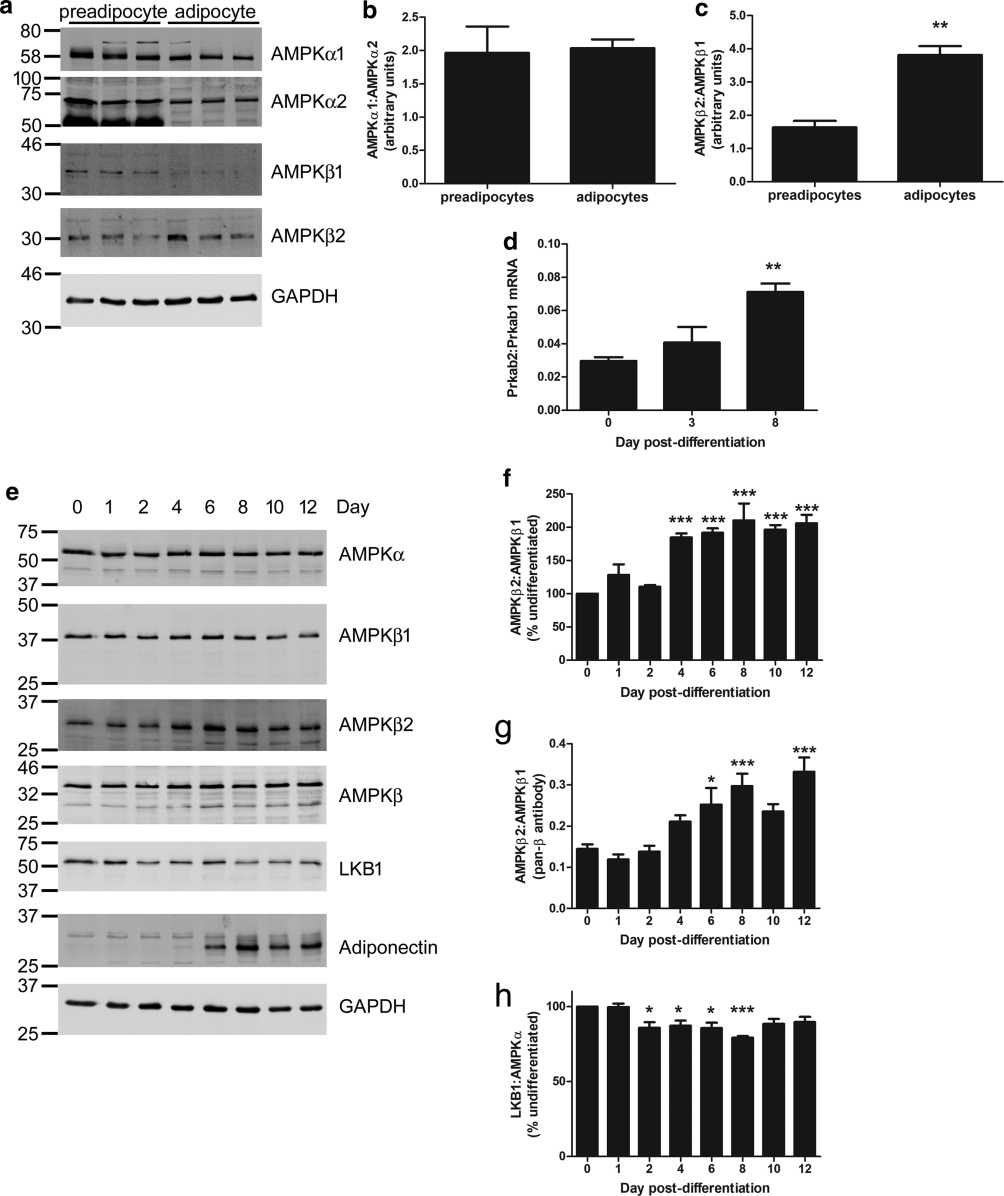
AMPKβ2 levels increase during 3T3-L1 cell adipogenesis. (**a**–**c**) 3T3-L1 preadipocytes were differentiated into adipocytes over 12 days and lysates prepared. Representative immunoblots are shown a) with the molecular masses (in kDa) indicated. Quantification of (**b**) AMPKα1:AMPKα2 or (**c**) AMPKβ2:AMPKβ1, assessed using individual isoform-specific antibodies. (**d**–**h**) 3T3-L1 preadipocytes were differentiated into adipocytes and (**d**) RNA or (**e**–**h**) lysates prepared at the indicated times during adipogenesis. (**d**) mRNA levels were assessed by qPCR and data shown represents Prkab2 (AMPKβ2) mRNA expression relative to Prkab1 (AMPKβ1). (**e**–**h**) Lysate proteins were resolved by SDS–PAGE and immunoblotted with the antibodies indicated. (**e**) Representative immunoblots are shown with the molecular masses (in kDa) indicated. Quantification of AMPKβ2:AMPKβ1 assessed using (**f**) individual isoform-specific antibodies or (**g**) antibodies recognising both AMPKβ isoforms and (**h**) LKB1:AMPKα levels over the duration of adipogenesis. Data is representative of three independent experiments, * *P *< 0.05, ** *P *< 0.01, *** *P *< 0.001 relative to preadipocyte levels (**c**) two-tailed *t*-test, (**d** and **f**–**h**) one-way ANOVA.

To examine whether levels of AMPK isoforms were altered during adipogenesis of 3T3-L1 adipocytes, levels of each AMPK subunit isoform were assessed by immunoblotting in 3T3-L1 preadipocytes and 12 days post-differentiation into adipocytes. There was no change in the relative proportion of AMPKα subunits, as assessed by the ratio of AMPKα1:AMPKα2 during adipogenesis (Figure 1a,b), nor was there any marked change in the total levels of AMPKα, as assessed with an antibody recognising both AMPKα1 and AMPKα2 (Figure 1e). In contrast, there was a marked 2-fold increase in the levels of AMPKβ2 relative to AMPKβ1 as 3T3-L1 preadipocytes were differentiated into adipocytes, observed by 4 days post-differentiation assessed with AMPKβ isoform-specific antibodies (Figure 1a,c,e,f). The increase in the AMPKβ2:AMPKβ1 protein ratio was associated with a significant increase in the levels of Prkab2 mRNA relative to Prkab1, yet levels of Prkab2 mRNA were ∼30-fold lower in preadipocytes and ∼15-fold lower in adipocytes relative to Prkab1 (Figure 1d). Furthermore, the increase in the relative ratio of Prkab2:Prkab1 was largely due to increased levels of Prkab2 when normalised to Tbp (Supplementary Figure S1). Similarly, when using an antibody that recognises both AMPKβ isoforms, AMPKβ2 immunoreactivity was 15% in preadipocytes and 32% in adipocytes relative to AMPKβ1 immunoreactivity (Figure 1e,g). Intriguingly, a small, but significant reduction in levels of the AMPK kinase, LKB1, was also observed relative to levels of AMPKα, during days 2–8 of 3T3-L1 adipogenesis (Figure 1e,h).

**Figure 2.**
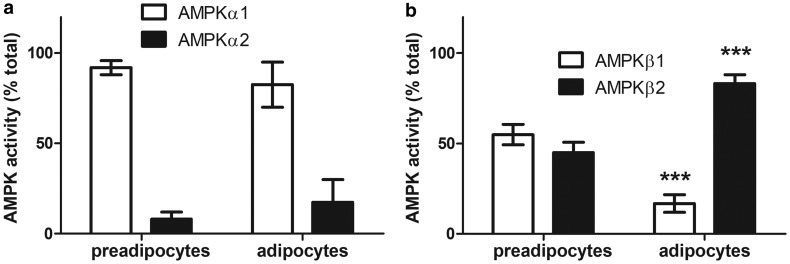
Isoform-specific AMPK activity in 3T3-L1 preadipocytes and adipocytes. AMPK was immunoprecipitated in lysates (100 µg) from 3T3-L1 preadipocytes or adipocytes using (**a**) antibodies specific to AMPKα1 or AMPKα2 or (**b**) antibodies specific to AMPKβ1 or AMPKβ2 and AMPK activity assayed. The values are expressed as % total AMPK activity (AMPKα1 + AMPKα2 or AMPKβ1 + AMPKβ2, respectively). Results shown are from three (AMPKα isoforms in preadipocytes), four (AMPKα isoforms in adipocytes), five (AMPKβ isoforms in preadipocytes) or six (AMPKβ isoforms in adipocytes) independent experiments. *** *P *< 0.001 relative to % activity in preadipocytes (unpaired *t*-test).

**Figure 3.**
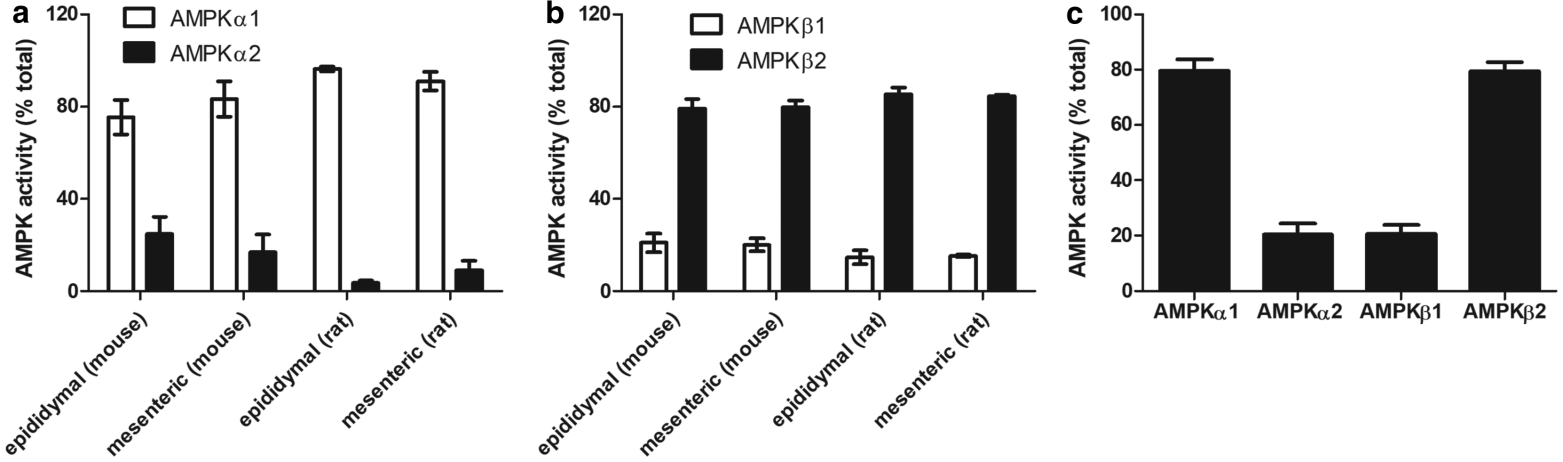
Isoform-specific AMPK activity in rodent adipocytes and human subcutaneous adipose tissue. AMPK was assayed in AMPK isoform-specific immunoprecipitates of lysates (100 µg) prepared from (**a** and **b**) rodent adipocytes isolated from mesenteric or epididymal adipose tissue or (**c**) human subcutaneous adipose tissue. Results shown are from five (mouse and rat epididymal adipocytes), four (mouse mesenteric adipose tissue), three (rat mesenteric adipocytes) or 10 (human subcutaneous adipose tissue) individual rodents or volunteers.

The specific total AMPK activity did not significantly change as 3T3-L1 preadipocytes differentiated into adipocytes (Supplementary Figure S2). Furthermore, levels of phosphorylation of the AMPK substrate, ACC relative to total ACC did not alter during adipogenesis, although levels of both phosphorylated and total ACC immunoreactivity increased during adipogenesis relative to total cellular protein levels (Supplementary Figure S2). Assays of AMPK complexes containing specific α- and β-subunit isoforms immunoprecipitated from 3T3-L1 preadipocytes and adipocytes confirmed that there was no change in AMPKα subunit isoforms as 3T3-L1 cells differentiated from preadipocytes to adipocytes, with complexes containing AMPKα1 representing 92 ± 4% and 83 ± 13% of the total immunoprecipitated AMPKα activity, respectively (Figure 2a). In agreement with the immunoblotting data, there was a marked increase in the total cellular AMPK activity of complexes containing AMPKβ2 during adipogenesis of 3T3-L1 adipocytes (Figure 2b), yet surprisingly complexes containing AMPKβ2 accounted for 45 ± 6% of the total immunoprecipitated AMPKβ activity in preadipocytes, increasing to 83 ± 5% of the activity in 3T3-L1 adipocytes (Figure 2b). Complexes containing AMPKα1 and AMPKβ2 also accounted for the majority of total AMPKα or AMPKβ immunoprecipitated activity in isolated adipocytes from two WAT depots (mesenteric and epididymal) obtained from both rats and mice (Figure 3a,b), indicating that 3T3-L1 adipocytes phenocopy the AMPK subunit isoform distribution in primary adipocytes. To determine whether this distribution of isoforms was also the case in human adipose tissue, the contribution of complexes containing particular AMPKβ and AMPKα isoforms to total AMPK activity was also assessed in human SCAT lysates obtained previously with ethical approval [35]. Similar to 3T3-L1 adipocytes and isolated rodent adipocytes, complexes containing AMPKα1 or AMPKβ2 accounted for 80 ± 4% and 79 ± 3% of total immunoprecipitated AMPKα or AMPKβ activity, respectively, in human SCAT (Figure 3c). To ensure that the low levels of activity associated with complexes containing AMPKα2 and AMPKβ1 were quantitative and not due to inefficient measurement of activity in immunoprecipitates, AMPK activity was measured in both immunoprecipitates and immunodepleted samples from mouse liver, which has previously been reported to contain high levels of AMPKβ1 and AMPKα2 [36]. AMPK activity in anti-AMPKα1 and anti-AMPKα2 immunoprecipitates accounted for 98% of total AMPK activity (40.8 and 57.7%, respectively), whereas AMPK activity in anti-AMPKβ1 and anti-AMPKβ2 immunoprecipitates accounted for 70% of total AMPK activity (59.2 and 11.1%, respectively; Supplementary Table S1). Recovery of AMPK activity in immunoprecipitates and immunodepleted samples combined was 84–98% relative to the original liver lysate (Supplementary Table S1). Furthermore, the combined activity immunoprecipitated from 3T3-L1 cells with anti-AMPKβ1 and anti-AMPKβ2 antibodies was 74.5 ± 7.0% in preadipocytes or 104.7 ± 41.0% in adipocytes (mean ± SEM, *n* = 3 in each case) of the combined activity immunoprecipitated with anti-AMPKα1 and anti-AMPKα2 antibodies. These data indicate that AMPK activity may be a partial, but not a substantive underestimate in AMPKβ1 immunoprecipitates of the actual activity.

**Figure 4.**
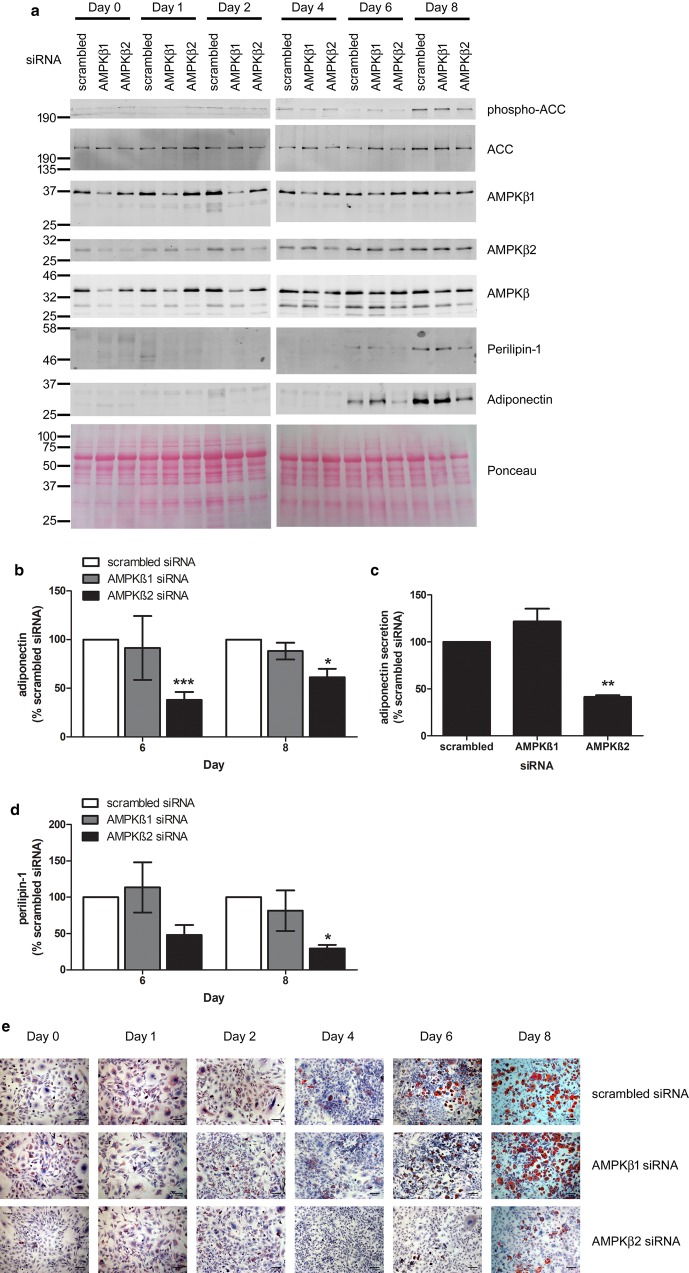
Down-regulation of AMPKβ2 suppresses adiponectin levels and lipid accumulation in 3T3-L1 adipocytes. 3T3-L1 preadipocytes were incubated with siRNA targeted to AMPKβ1, AMPKβ2 or scrambled siRNA for 48 h prior to differentiation into adipocytes. (**a**, **b** and **d**) Lysates were prepared after the indicated durations after initiation of differentiation and resolved by SDS–PAGE and immunoblotting with the indicated antibodies. (**a**) Representative immunoblots are shown, repeated on two to five further occasions. Densitometric analysis of (**b**) adiponectin or (**d**) perilipin-1 levels (days 6 and 8), normalised to Ponceau staining. (**c**) Conditioned media was collected from 3T3-L1 adipocytes at day 8 post-differentiation and adiponectin measured by ELISA in three independent experiments. (**e**) Cells were fixed and stained with oil red O at the times indicated during differentiation. Representative images from three independent experiments are shown, scale bar represents 20 µm. * *P* < 0.05, ** *P* < 0.01, *** *P* < 0.001 relative to scrambled siRNA (one-way ANOVA).

As there was a clear change in the levels and activity of complexes containing AMPKβ isoforms during adipogenesis of 3T3-L1 cells, the effect of down-regulation of either AMPKβ1 or AMPKβ2 isoform was assessed on the differentiation of 3T3-L1 preadipocytes. Incubation with siRNA targeting AMPKβ1 successfully down-regulated levels of AMPKβ1 by 70 ± 7% after 48 h (day 0) in 3T3-L1 preadipocytes without altering levels of AMPKβ2 (Figure 4a and Supplementary Figure S3). Similarly, siRNA targeting AMPKβ2 successfully down-regulated levels of AMPKβ2 by 52 ± 9% without altering levels of AMPKβ1 (Figure 4a and Supplementary Figure S3). Down-regulation of AMPKβ1 or AMPKβ2 tended to reduce ACC phosphorylation when compared with scrambled control siRNA, yet this did not achieve statistical significance except with siRNA targeted to AMPKβ1 at day 1 (Figure 4a and Supplementary Figure S3). Adiponectin, which maintains healthy adipose tissue expansion and regulates whole-body insulin sensitivity [37], is expressed and secreted by mature adipocytes. Adiponectin immunoreactivity was observed in scrambled siRNA-treated 3T3-L1 cells 6–8 days after the addition of adipogenic stimuli (Figure 4a,b). Down-regulation of AMPKβ1 in preadipocytes had no effect on cellular levels of adiponectin 6–8 days after stimulation of adipogenesis, yet down-regulation of AMPKβ2 markedly reduced adiponectin levels 6–8 days post stimulation (Figure 4b). Furthermore, adiponectin levels in conditioned medium taken from cells 8 days after stimulation of differentiation were markedly lower in medium from 3T3-L1 cells originally incubated with AMPKβ2 siRNA, whereas AMPKβ1 siRNA had no effect (Figure 4c). Similar to adiponectin, down-regulation of AMPKβ2, but not AMPKβ1 markedly reduced induction of the lipid droplet-associated protein, perilipin-1 in later stages of adipogenesis (Figure 4d). Furthermore, in 3T3-L1 preadipocytes incubated for 48 h with siRNA targeted to AMPKβ2, there was markedly less oil red O staining 8 days after the addition of adipogenic stimuli, whereas cells incubated with siRNA targeting AMPKβ1 exhibited similar lipid accumulation over time as those incubated with scrambled siRNA (Figure 4e). It is worth noting that although cells were only incubated with siRNA for 48 h prior to stimulation of differentiation with insulin, dexamethasone and IBMX, when the media was removed, down-regulation of AMPKβ1 and AMPKβ2 was observed for up to 4 days after stimulation of differentiation, 6 days after the initial incubation with siRNA (Figure 4a and Supplementary Figure S3).

**Figure 5.**
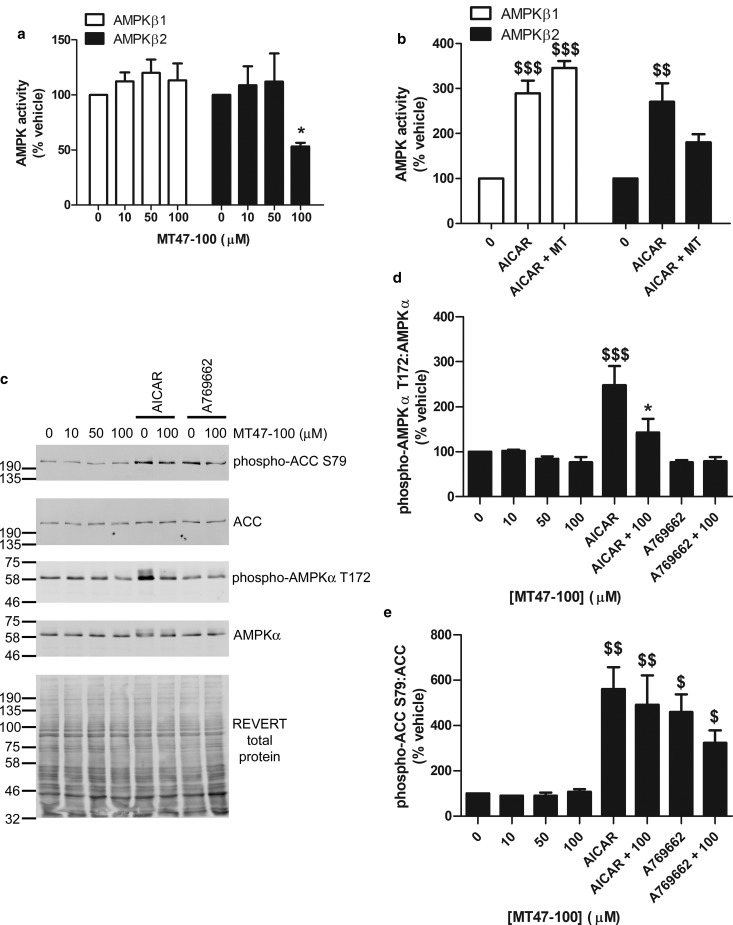
MT47-100 inhibits AMPK activity of complexes containing AMPKβ2 and suppresses AICAR-stimulated AMPKα T172 phosphorylation in 3T3-L1 preadipocytes. 3T3-L1 preadipocytes were incubated in the presence or absence of the indicated concentrations of MT47-100 for (**a**) 1 h or (**b**–**e**) 30 min prior to incubation in the presence or absence of AICAR (2 mmol/l) or A769662 (100 µmol/l) for 1 h and cell lysates prepared. (**a** and **b**) AMPKβ1 or AMPKβ2 were immunoprecipitated from lysates and AMPK activity subsequently assessed in immunoprecipitates. Data shown are from (**a**) five or (**b**) three independent experiments. (**c**–**e**) Lysate proteins were resolved by SDS–PAGE and immunoblotted with the indicated antibodies. (**c**) Representative immunoblots are shown, repeated on two further occasions. Densitometric analysis of (**d**) phospho-AMPKα or (**e**) phospho-ACC relative to AMPKα and ACC, respectively. *P < 0.05 relative to absence of MT47-100, ^$^*P* < 0.05, ^$$^*P* < 0.01, ^$$$^*P* < 0.001 relative to absence of AICAR or A769662 (one-way ANOVA).

To further confirm the important role of AMPK complexes containing AMPKβ2 in lipid accumulation and adiponectin secretion, we incubated 3T3-L1 preadipocytes with the dihydroxyquinoline MT47-100, which has previously been demonstrated to selectively inhibit AMPKβ2-containing complexes [34]. Incubation of 3T3-L1 preadipocytes with 100 µmol/l MT47-100, a concentration previously demonstrated to inhibit complexes containing AMPKβ2 in isolated hepatocytes [34], inhibited the activity of immunoprecipitated complexes containing AMPKβ2 47 ± 3%, without influencing the activity of immunoprecipitated AMPKβ1 complexes (Figure 5a). Concentrations lower than 100 µmol/l had no effect on AMPK activity in complexes containing either isoform (Figure 5a). Incubation with AICAR, which is phosphorylated to the AMP mimetic ZMP within cells, thereby activating all AMPK complexes, markedly stimulated the activity of immunoprecipitated complexes containing AMPKβ1 or AMPKβ2 in 3T3-L1 preadipocytes, yet the statistical significance of this stimulation in complexes containing AMPKβ2 was absent upon preincubation with MT47-100 (Figure 5b). MT47-100 had no significant effect on basal AMPKα T172 phosphorylation, yet impaired AICAR-stimulated AMPKα T172 phosphorylation (Figure 5c,d). Intriguingly, despite this inhibition of AMPKα T172 phosphorylation, MT47-100 had no effect on basal or AICAR-stimulated phosphorylation of the AMPK substrate ACC (Figure 5c,e). Furthermore, as expected, MT47-100 had no effect on ACC phosphorylation stimulated by the AMPKβ1-selective activator A769662 (Figure 5c,e).

**Figure 6.**
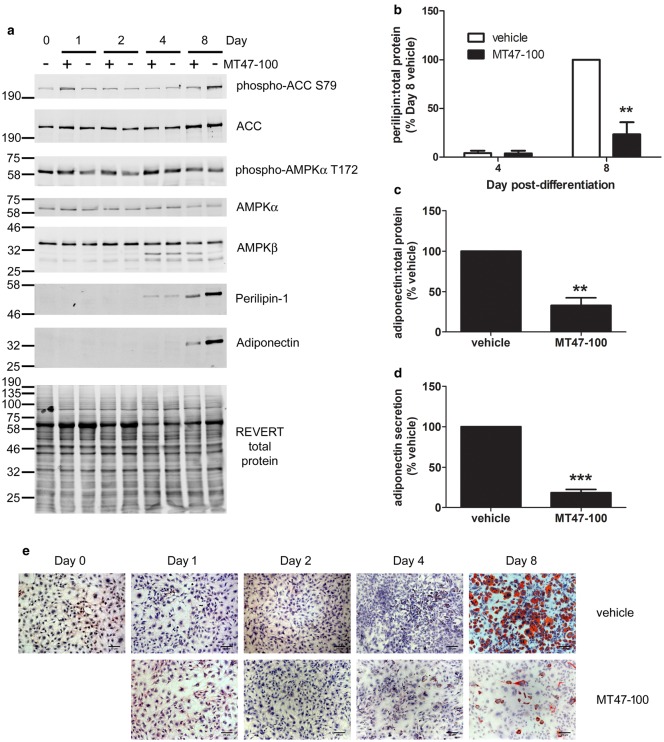
MT47-100 suppresses adiponectin secretion and lipid accumulation during adipogenesis. 3T3-L1 preadipocytes were differentiated in the presence or absence of MT47-100 (100 µmol/l), with media changed on days 3 and 6, when MT47-100 was re-introduced to the culture medium. (**a**–**c**) Cell lysates were prepared at the times indicated and proteins resolved by SDS–PAGE and immunoblotting with the antibodies indicated. (**a**) Representative immunoblots, repeated with similar results on two further occasions are shown. Densitometric analysis of (**b**) perilipin-1 or (**c**) adiponectin levels relative to REVERT total protein stain at 8 days post-differentiation from three independent experiments. (**d**) Conditioned media were collected from 3T3-L1 adipocytes differentiated in the absence or presence of MT47-100 (100 µmol/l) for 8 days and adiponectin measured by ELISA in three independent experiments. (**e**) Cells were fixed and stained with oil red O at the times indicated during differentiation. Representative images from three independent experiments are shown, scale bar represents 20 µm. ** *P* < 0.01, *** *P* < 0.001 relative to absence of MT47-100 (**b**) 2-way ANOVA (**c** and **d**) unpaired *t*-test.

As MT47-100 inhibited complexes containing AMPKβ2 in preadipocytes, the effect of MT47-100 on adipogenesis was examined. Differentiation of 3T3-L1 preadipocytes in the presence of MT47-100 abrogated induction of perilipin-1 and adiponectin protein levels by day 8 (Figure 6a–c), with concomitant inhibition of adiponectin secretion into the cell medium (Figure 6d) and lipid accumulation (Figure 6e). MT47-100 had no significant effect on AMPKα or ACC phosphorylation on any day during adipogenesis except a marked inhibition of ACC phosphorylation at day 8 (Figure 6a and Supplementary Figure S4). Furthermore, MT47-100 had no effect on the change of expression of AMPKβ2:AMPKβ1 during adipogenesis (Figure 6a and Supplementary Figure S4).

**Figure 7.**
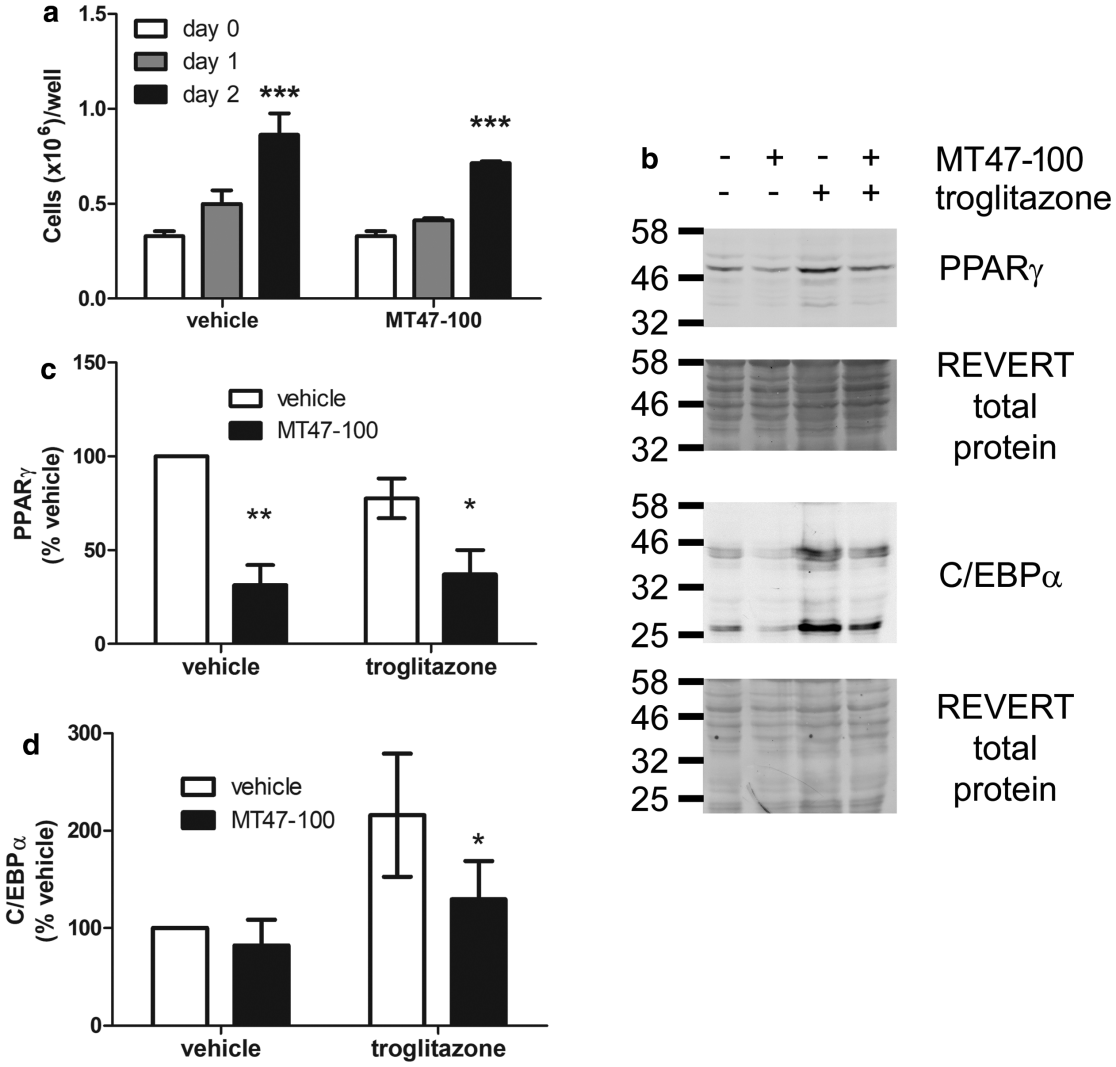
MT47-100 inhibits cellular PPARγ and C/EBPα levels during differentiation of 3T3-L1 cells. (**a**) 3T3-L1 preadipocytes in six-well plates were differentiated in the presence of troglitazone (1 µmol/l) and the absence or presence of MT47-100 (100 µmol/l), trypsinised and counted using a Nexcelom Cellometer Auto 1000 on the days indicated after commencement of differentiation. Data shown represent the mean ± SEM cells/well from three independent experiments. *** *P* < 0.001 relative to day 0 (two-way ANOVA). (**b**–**d**) 3T3-L1 preadipocytes were differentiated in the absence or presence of MT47-100 (100 µmol/l) as well as troglitazone (1 µmol/l) and cell lysates were prepared 3 days post-differentiation and equal volumes resolved by SDS–PAGE and immunoblotting with the antibodies indicated. (**b**) Representative immunoblots, repeated with similar results on two further occasions are shown. (**c** and **d**) Densitometric analysis of protein levels relative to total lysate protein, assessed with REVERT total protein stain (Li-Cor Biosciences). Data shown represents the mean ± SEM relative to vehicle control. * *P* < 0.05, ** *P* < 0.01 relative to absence of MT47-100 (two-way ANOVA).

Adipogenesis in 3T3-L1 adipocytes involves confluent preadipocytes re-entering the cell cycle and undergoing several rounds of division called mitotic clonal expansion (MCE), during which there is induction of the key adipogenic transcription factors PPARγ and C/EBPα [14], which subsequently lead to the expression of lipogenic proteins and adiponectin. Differentiation of 3T3-L1 preadipocytes was associated with an approximate doubling of the cell number over the first 2 days, an effect that was not significantly altered by co-incubation in the presence of 100 µmol/l MT47-100 (Figure 7a). Furthermore, co-incubation of 3T3-L1 cells with MT47-100 during differentiation in the absence of troglitazone reduced PPARγ levels after 3 days yet had no effect on C/EBPα levels (Figure 7b–d). In cells differentiated in the presence of troglitazone, co-incubation with MT47-100 reduced levels of both PPARγ and C/EBPα (Figure 7b–d).

## Discussion

These data clearly demonstrate that AMPKα1β2 complexes are the principal isoforms at the level of total cellular activity in rodent adipocytes and human adipose tissue. Furthermore, this phenotype is conserved during adipogenesis in 3T3-L1 cells, when there is a change in expression from complexes containing AMPKα1β1 to those containing AMPKα1β2. Suppression of AMPKβ2 activity in 3T3-L1 preadipocytes by siRNA-mediated down-regulation or with MT47-100 abrogates adipogenesis, suggesting that the switch from complexes containing AMPKβ1 to complexes containing AMPKβ2 is important for efficient adipogenesis in 3T3-L1 adipocytes.

The demonstration that AMPKα1 is the principal catalytic subunit isoform in terms of total cellular AMPK activity in 3T3-L1 adipocytes, isolated rodent adipocytes and human SCAT agrees with previous reports [38–41]. Furthermore, AMPK complexes containing β2 account for the majority of total cellular AMPK activity in mouse and rat adipocytes isolated from two separate WAT depots as well as human SCAT, phenocopying 3T3-L1 adipocytes and suggesting the switch from complexes containing AMPKβ1 to those containing AMPKβ2 during adipogenesis in 3T3-L1 cells reflects normal physiology.

Prior to this study, little was known concerning the role of AMPKβ isoforms in adipocyte function or adipose tissue physiology. This substantial contribution of AMPKβ2 to total AMPK levels in adipocytes is in agreement with the observation that AMPK activity is reduced by only ∼30% in WAT of mice lacking AMPKβ1 [42]. Surprisingly, however, the activity of AMPKα1 or AMPKα2 in WAT of mice lacking AMPKβ2 has been reported to be unchanged [43], suggesting adipocytes in those mice may exhibit compensatory increases in AMPKβ1 levels. In contrast with the kinase assay data showing AMPK complexes containing β2 account for the majority of total cellular AMPK activity in adipocytes, there was 7-fold or 3-fold greater AMPKβ1 immunoreactivity relative to AMPKβ2 in preadipocytes and adipocytes, respectively, when assessed using an antibody that recognises both AMPKβ isoforms. Even accounting for the potential underestimation of AMPK activity in AMPKβ1 immunoprecipitates, complexes containing AMPKβ2 will represent the major activity in 3T3-L1 adipocytes, rodent adipocytes and human SCAT. This suggests either that the antibody that recognises both AMPKβ isoforms does not exhibit equal affinity for each isoform or that the specific activity of complexes containing AMPKβ2 is far greater than those containing AMPKβ1. During the preparation of this manuscript, it was reported that the levels of AMPKβ2 relative to AMPKβ1 do not change between 3T3-L1 preadipocytes and 3T3-L1 adipocytes using the same antibody, although mouse and human adipocytes exhibited substantial levels of AMPKβ2 immunoreactivity [41]. The reasons for this discrepancy are uncertain. Furthermore, the reason for the increase in AMPKβ2:AMPKβ1 during adipogenesis is likely to reflect altered transcription of Prkab2 and Prkab1, respectively, as there was a concomitant increase in Prkab2 mRNA during adipogenesis. Intriguingly, Prkab1 mRNA was expressed at a substantially higher level than Prkab2 mRNA.

Adipogenesis in 3T3-L1 adipocytes involves MCE, during which PPARγ and C/EBPα are induced [14], leading to the expression of lipogenic proteins and adiponectin. Numerous previous studies have reported that AMPK activation inhibits differentiation of preadipocytes [14,16–21], whereas down-regulation of AMPK increases the extent of adipogenesis in preadipocytes [23–27]. The mechanism by which AMPK activation inhibits adipogenesis includes early suppression of MCE and expression of PPARγ and C/EBPα, whilst maintaining levels of Pref-1 (preadipocyte factor 1), which is normally suppressed during early adipogenesis [14–19,21,24,26,27]. The inhibition of PPARγ expression by AMPK activation has further been reported to be mediated by stimulation of Wnt/β-catenin signalling [17,44].

In contrast with these studies, the current study suggests that expression of AMPKβ2 is required for efficient adipogenesis of 3T3-L1 adipocytes. Two independent lines of evidence support this. Firstly, down-regulation of AMPKβ2, but not AMPKβ1 in 3T3-L1 preadipocytes markedly attenuated lipid accumulation as well as levels and secretion of the marker of late adipogenesis, adiponectin. These data suggest that the increase in AMPKβ2 levels is required for efficient adipogenesis, yet the suppression of AMPKβ1 does not increase the adipogenic potential of the preadiocytes, indicating that increased AMPKβ2 and not decreased AMPKβ1 underlies the effect on adipogenesis. Secondly, MT47-100, which inhibited AMPKβ2-containing complexes without altering the activity of AMPKβ1-containing complexes markedly inhibited both lipid accumulation and adiponectin levels and secretion, phenocopying the effect of AMPKβ2-targeted siRNA. Interestingly, the switch from AMPKβ1 to AMPKβ2 occurred early (days 2–4) during adipogenesis, suggesting an early effect regulated by adipogenic transcription factors. Furthermore, inhibition of complexes containing AMPKβ2 with MT47-100 was associated with suppression of PPARγ and C/EBPα levels, without altering cell number during MCE, suggesting that complexes containing AMPKβ2 contribute to increased adipogenic transcription factor expression at this point.

These data are supported by previous studies using the poorly-selective AMPK inhibitor, compound C, which has also been demonstrated to inhibit adipogenic gene expression, MCE and lipid accumulation during adipogenesis in 3T3-L1 cells and CH3H10T1/2 mesenchymal stem cells [19,45,46], yet the AMPK-dependence of this effect was not demonstrated in those studies and compound C has also been reported to increase adipogenic gene expression in human adipose tissue mesenchymal stem cells [24]. We and others have previously reported that incubation of mouse embryonic fibroblasts (MEFs) lacking AMPKα1 and AMPKα2 with adipogenic stimuli failed to increase lipid accumulation compared with wild type MEFs, further suggesting that some AMPK activity is required for adipogenic gene expression [19,47], which has also been observed in MEFs lacking LKB1 [47].

MT47-100 inhibited AMPKβ2 complexes without altering AMPKβ1 complex activity in unstimulated and AICAR-stimulated 3T3-L1 preadipocytes, as assessed with assays of immunoprecipitated AMPK which reflect activity due to covalent modification of AMPK. Despite this, MT47-100 had no significant effect on phospho-AMPKα Thr172 levels in unstimulated 3T3-L1 preadipocytes, but did inhibit AICAR-stimulated Thr172 phosphorylation. Furthermore, MT47-100 was only effective at a concentration that has previously been demonstrated to inhibit AMPKβ2 complexes and allosterically stimulate AMPKβ1-containing complexes in isolated mouse hepatocytes [34]. It remains entirely possible that either MT47-100 or siRNA targeted to AMPKβ complexes have off-target effects that might influence adipogenesis, yet the marked correlation between these two divergent but complimentary methods of down-regulating AMPKβ2 strongly argue against this effect being independent of AMPKβ2.

We and others have reported that the AMPK activator, A769662, which selectively activates AMPK complexes containing the β1 isoform [48], inhibits adipogenesis in 3T3-L1 cells and CH3H10T1/2 mesenchymal stem cells [16,19,20], suggesting that AMPKβ1 activation suppresses adipogenesis. Although an AMPKβ1-mediated effect of MT47-100 cannot be completely ruled out, down-regulation of AMPKβ1 using siRNA had no effect on adipogenesis in 3T3-L1 cells, such that the effect of MT47-100 is far likelier to be mediated by AMPKβ2 inhibition, as it phenocopies siRNA-mediated AMPKβ2 down-regulation.

Mice lacking AMPKα1 exhibit reduced adipose tissue mass and smaller adipocytes [40]. Similarly, mice with a global deletion of AMPKβ1 exhibit reduced fat mass [42], yet no WAT phenotype has been reported in mice that lack AMPKβ2 [43], although they have been reported to exhibit increased susceptibility to adiposity in response to a high fat diet [49]. Whether impaired adipogenesis contributes to the adipose phenotypes of AMPKα1 or AMPKβ1 knockout mice has not been assessed, as it is compromised by the multiple effects of AMPK on carbohydrate and lipid metabolism, as well as feeding behaviour. More recently, mice with adipose-targeted deletion of AMPK subunit isoforms have been developed [50–52], yet they have utilised the adiponectin promoter to drive recombination in mature adipocytes, rather than preadipocytes, such that early adipogenesis would likely be unaffected by genetic alterations in those mice.

Taken together, the data presented in this study demonstrate a hitherto unappreciated role for AMPK complexes containing AMPKβ2, to improve the efficiency of early adipogenesis of 3T3-L1 adipocytes. The mechanisms underlying this action of AMPKβ2 complexes containing AMPKβ2 remain uncertain, yet likely involve improvements in PPARγ- and C/EBPα-mediated transcription after the MCE stage.

## Abbreviations

ACC: acetyl-CoA carboxylase
AMPK: AMP-activated protein kinase
BAT: brown adipose tissue
C/EBPα: CCAAT-enhancer-binding protein-α
CaMKK2: Ca^2+^-calmodulin-dependent kinase kinase-2
GAPDH: glyceraldehyde-3-phosphate dehydrogenase
HFD: high fat diet
IBMX: 3-isobutyl-1-methylxanthine
LKB1: liver kinase B1
MCE: mitotic clonal expansion
MEFs: mouse embryonic fibroblasts
NAFLD: non-alcoholic fatty liver disease
NCS: newborn calf serum
PEG: polyethylene glycol
PPARγ: peroxisome proliferator-activated receptor-γ
SCAT: subcutaneous adipose tissue
Tbp: TATA-binding protein mRNA
WAT: white adipose tissue
