# Supplementary material for: AMP-activated protein kinase complexes containing the β2 regulatory subunit are up-regulated during and contribute to adipogenesis
Source: Biochem J. 2019 Jun 26;476(12):1725–40. doi: 10.1042/BCJ20180714 (PMC6595317; doi:10.1042/BCJ20180714)
Supplement: Supplementary Figures and Table [file BCJ-476-1725-s1.pdf]

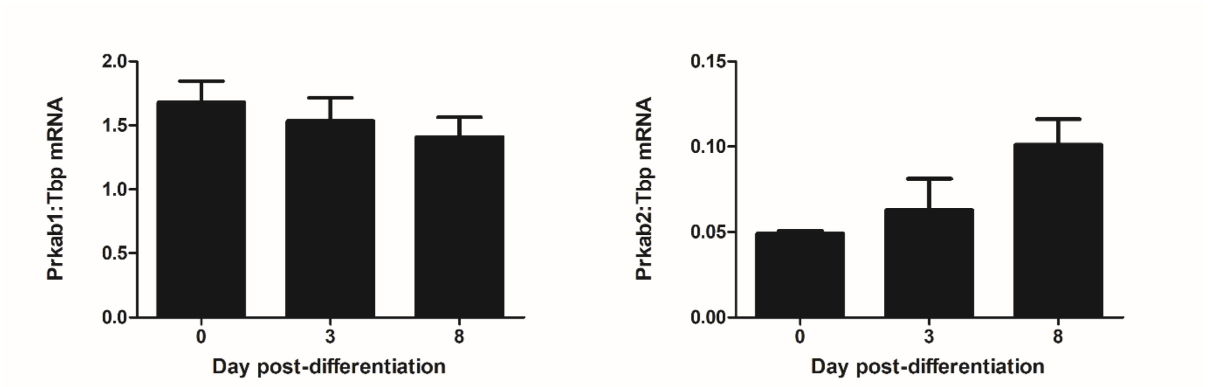

Supplementary Figure 1: AMPKβ1 and AMPKβ2 mRNA levels during 3T3-L1 cell adipogenesis.

3T3-L1 preadipocytes were differentiated into adipocytes and RNA prepared at the indicated times during adipogenesis. mRNA levels were assessed by qPCR and data shown represents Prkab2 (AMPKβ2) or Prkab1 (AMPKβ1) mRNA levels relative to Tbp. Data is representative of three independent experiments.

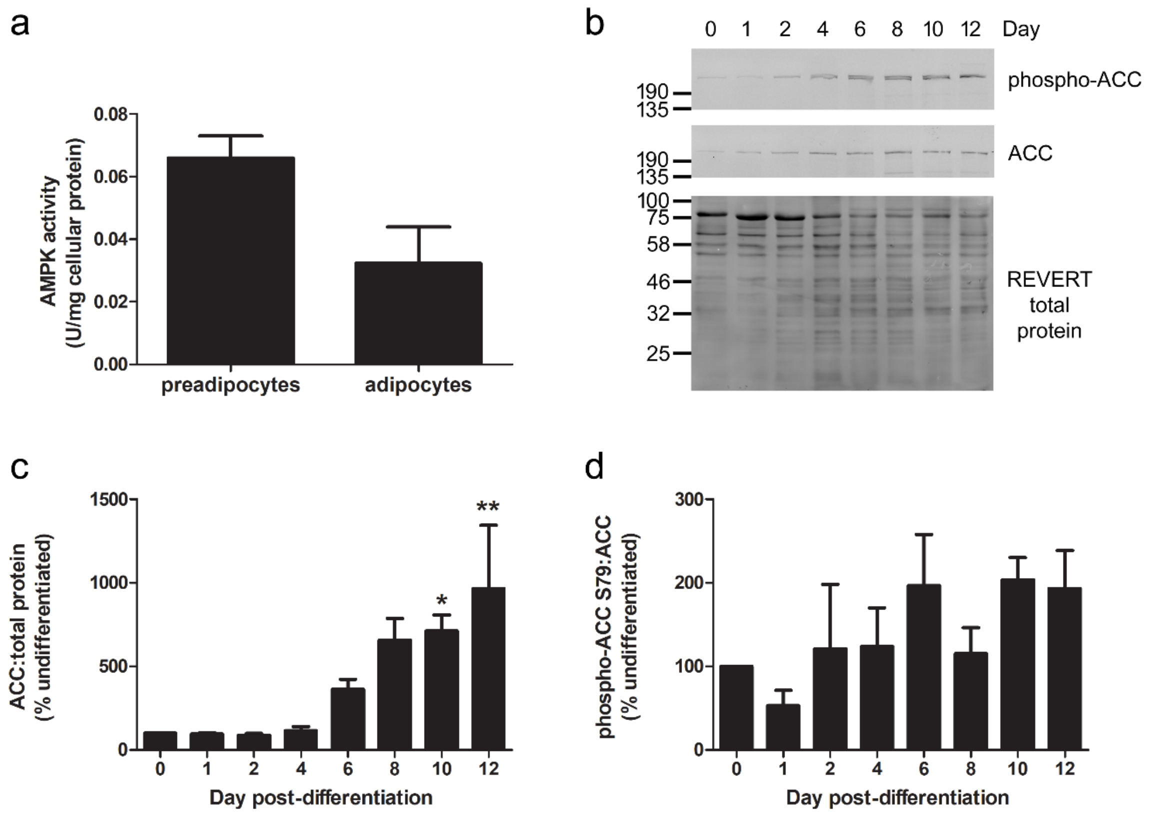

Supplementary Figure 2: AMPK activity during 3T3-L1 cell adipogenesis.

3T3-L1 preadipocytes were differentiated into adipocytes over 12 days and lysates prepared. a) Lysates from day 0 and day 12 were immunoprecipitated with anti-AMPKα1 and anti-AMPKα2 antibodies and AMPK activity assessed. Activity is expressed in U/mg lysate protein, where 1 U = 1 nmol/min ^{32}P incorporated from [^{32}P]ATP into SAMS peptide. b-d) Lysate proteins were resolved by SDS-PAGE and immunoblotted with the antibodies indicated. b) Representative immunoblots are shown with the molecular masses (in kDa) indicated. Quantification of c) ACC levels relative to total lysate protein, assessed with REVERT total protein stain or d) phospho-ACC relative to ACC levels over the duration of adipogenesis. Data is representative of three independent experiments, *P<0.05, ** P<0.01 relative to preadipocyte levels (one-way ANOVA).

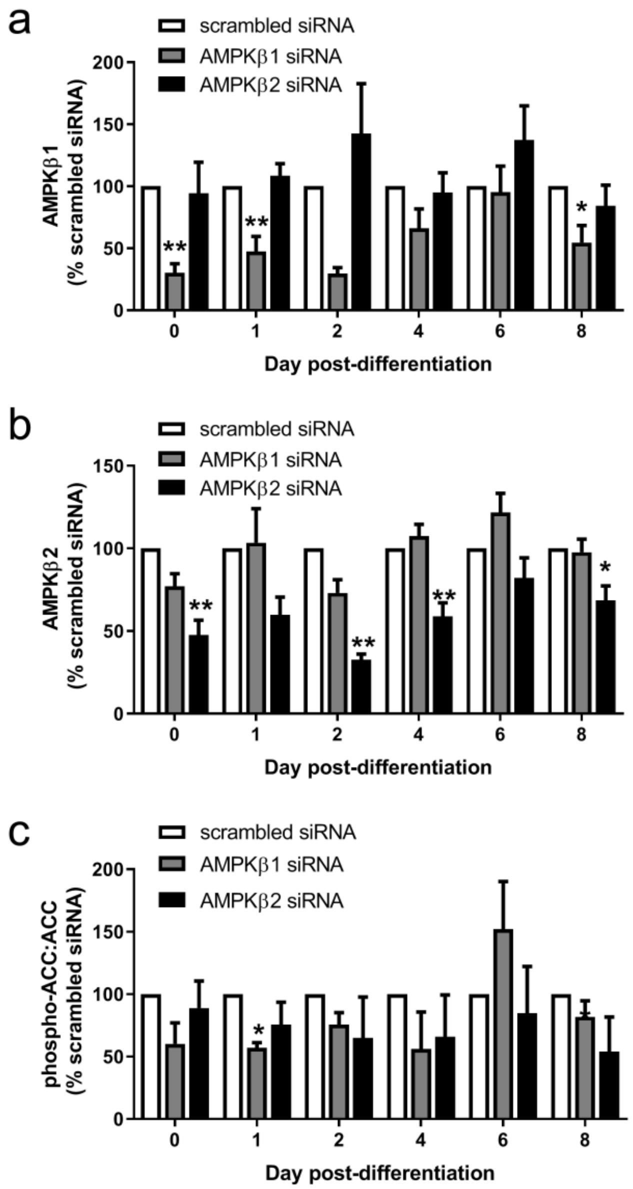

Supplementary Figure 3: Efficiency of siRNA-mediated downregulation of AMPKβ isoforms in 3T3-L1 preadipocytes.

3T3-L1 preadipocytes were incubated with siRNA targeted to AMPKβ1, AMPKβ2 or scrambled siRNA for 48 h prior to differentiation into adipocytes. Lysates were prepared after the indicated durations after initiation of differentiation and resolved by SDS-PAGE and immunoblotting with a) anti-AMPKβ1, b) anti-AMPKβ2 antibodies or c) anti-phospho-ACC S79 and anti-ACC antibodies. Densitometric analysis of the immunoblots shown in Figure 4, normalised to a, b) Ponceau staining from 6 independent experiments or c) ACC from 3 independent experiments. *$P<0.05$, **$P<0.01$ relative to scrambled siRNA (one-way ANOVA).

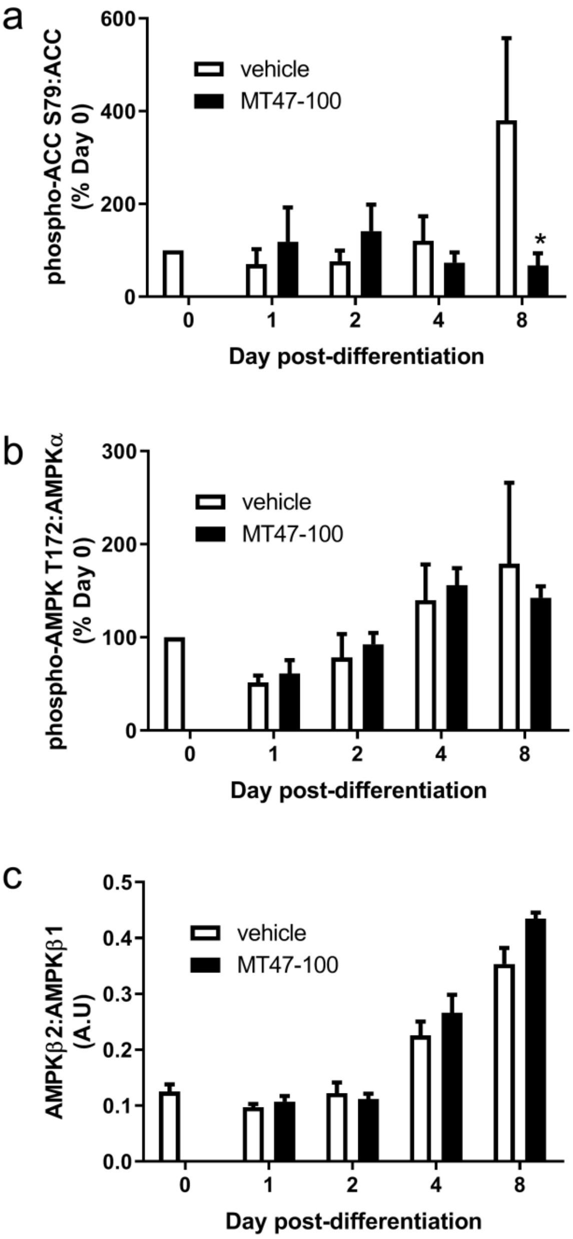

Supplementary Figure 4: Effect of MT47-100 on AMPKα and ACC phosphorylation during adipogenesis

3T3-L1 preadipocytes were differentiated in the presence or absence of MT47-100 (100 μmol/l), with media changed on days 3 and 6, when MT47-100 was re-introduced to the culture medium. Cell lysates were prepared at the times indicated and proteins resolved by SDS-PAGE and immunoblotting with a) anti-phospho-ACC S79 and anti-ACC antibodies, b) anti-phospho-AMPKα T172 and anti-AMPKα antibodies or c) anti-AMPKβ antibodies. Densitometric analysis of the immunoblots in Figure 6 is shown, from three independent experiments. *P<0.05 relative to absence of MT47-100 (2-way ANOVA).

Immunoprecipitated AMPK isoform	AMPK activity in IP (% IP + ID) (mean ± SEM)	% Recovery of AMPK activity in IP + ID relative to original sample (mean ± SD)
**AMPKα1**	**40.8 ± 2.9**	**91.8 ± 10.8**
**AMPKα2**	**57.7 ± 0.5**	**83.5 ± 3.7**
**AMPKβ1**	**59.2 ± 4.4**	**98.0 ± 7.3**
**AMPKβ2**	**11.1 ± 1.3**	**90.7 ± 6.5**

Supplementary Table 1: Efficiency of isoform-specific AMPK immunoprecipitation measurements

Mouse liver 2.5-6.25% PEG precipitates were prepared and resuspended in IP buffer. AMPK was immunoprecipitated using antibodies specific to AMPKα1, AMPKα2, AMPKβ1 or AMPKβ2 and AMPK activity assayed in the original resuspended PEG precipitates, immunoprecipitates (IP) and immunodepleted (ID) samples. Results shown are from three independent experiments.
